# A Multi-Modal Pelvic MRI Dataset for Deep Learning-Based Pelvic Organ Segmentation in Endometriosis

**DOI:** 10.1038/s41597-025-05623-3

**Published:** 2025-07-24

**Authors:** Xiaomin Liang, Linda A. Alpuing Radilla, Kamand Khalaj, Haaniya Dawoodally, Chinmay Mokashi, Xiaoming Guan, Kirk E. Roberts, Sunil A. Sheth, Varaha S. Tammisetti, Luca Giancardo

**Affiliations:** 1 McWilliams School of Biomedical Informatics, University of Texas Health Science Center at Houston, Houston, USA; 2 The Department of Obstetrics and Gynecology, Baylor College of Medicine, Houston, USA; 3 Texas Children’s Hospital Pavilion for Women, Houston, USA; 4 McGovern Medical School, University of Texas Health Science Center at Houston, Houston, USA; 5 Department of Diagnostic and Interventional Imaging, McGovern Medical School, University of Texas Health Science Center at Houston, Houston, USA

**Keywords:** Magnetic resonance imaging, Diseases

## Abstract

Endometriosis affects approximately 190 million females of reproductive age worldwide. Magnetic Resonance Imaging (MRI) has been recommended as the primary non-invasive diagnostic method for endometriosis. This study presents new female pelvic MRI multicenter datasets for endometriosis and shows the baseline segmentation performance of two auto-segmentation pipelines: the self-configuring nnU-Net and RAovSeg, a custom network. The multi-sequence endometriosis MRI scans from two clinical institutions were collected. A multicenter dataset of 51 subjects with manual labels for multiple pelvic structures from three raters was used to assess interrater agreement. A second single-center dataset of 81 subjects with labels for multiple pelvic structures from one rater was used to develop the ovary auto-segmentation pipelines. Uterus and ovary segmentations are available for all subjects, endometrioma segmentation is available for all subjects where it is detectable in the image. This study highlights the challenges of manual ovary segmentation in endometriosis MRI and emphasizes the need for an auto-segmentation method. The dataset is publicly available for further research in pelvic MRI auto-segmentation to support endometriosis research.

## Background & Summary

According to key facts released by the World Health Organization (WHO) in 2023, endometriosis affects approximately 190 million females of reproductive age worldwide^1^. The prevalence is often considered to be underestimated because the gold standard for diagnosing endometriosis relies on a surgical procedure called laparoscopy, which is not a routine examination^2,3^. Typically, only hospitalized female patients experiencing related symptoms such as chronic pelvic pain, abnormal cramping, and bleeding may undergo this procedure^4^. Ultrasound and Magnetic Resonance Imaging (MRI) have been recommended as the primary non-invasive diagnostic methods for endometriosis^5^. MRI can achieve over 90% diagnostic sensitivity and specificity in most cases^6,7^. An endometrioma on the ovary is a cystic lesion, affecting 17–44% of women diagnosed with endometriosis^8^. It can lead to infertility in some patients. Therefore, precise ovary segmentation based on three-dimensional (3D) MRI for endometriosis patients is crucial for endometrioma detection, surgical guidance, and predicting post-operative complications. In endometriosis patients, the ovary may be deformed or absent due to surgical resection, making this segmentation task challenging and underscoring the importance of experienced clinicians.

Recent studies have demonstrated the effectiveness of deep learning methods in pelvic organ segmentation using MRI, particularly for prostate cancer and cervical cancer^9–12^. A recent study explored a U-Net-based ensemble method for the endometriotic lesions segmentation using ultrasound^13^. However, automatic ovary segmentation methods for endometriosis patients based on MRI are scarce. Given the advancements in deep learning algorithms for medical imaging segmentation, an automatic segmentation pipeline for endometriosis MRI would be highly beneficial. Such a pipeline could reduce the manual labeling workload for clinicians and help standardize ovary segmentation for endometriosis, thereby minimizing inter-rater disagreement. The residual learning network, ResNet, is a highly influential deep convolutional network that has achieved high accuracy in disease diagnosis and organ detection based on MRI^14,15^. Attention U-Net, known for its stability and practical utility, is widely adopted in medical image segmentation applications^16,17^. Typically, training an auto-segmentation model requires an annotated dataset. Even though some recent studies based on the Segment Anything model have achieved advancements in medical imaging auto-segmentation, their methods require manually selected bounding boxes as prompts or extensive fine-tuning to effectively utilize text prompts relevant to this problem, making it challenging to achieve excellent performance with a limited dataset size^18–20^. Therefore, another U-Net-based model, nnU-Net, recognized as a state-of-the-art approach for various medical image segmentation tasks, has been widely adopted as a baseline for automatic segmentation^21,22^.

In this study, we constructed two endometriosis MRI datasets: a multicenter dataset and a single-site dataset, sourced from two distinct clinical institutions. The first endometriosis MRI dataset, which includes multi-rater annotations of pelvic organs, was used to assess inter-rater agreement and establish a baseline for ovary segmentation performance by comparing human raters with a state-of-the-art automatic segmentation method. In addition, the second dataset can serve as the foundation for developing the auto-segmentation method. Utilizing this dataset, we propose an automatic ovary segmentation pipeline, RAovSeg, for endometriosis patients by combining a ResNet-based classifier with an Attention U-Net-based segmentation network to ensure the dataset is suitable for developing an auto-segmentation method. We also adopted the nnU-Net as the baseline model.

This pipeline can aid in detecting and segmenting the ovary for endometriosis treatment, including surgical guidance. It highlights the importance of observing ovarian abnormalities in patients with superficial endometriosis and assists in predicting post-operative complications when an ovary is resected. Given the current lack of publicly accessible datasets for endometriosis MRI, this dataset will be a valuable resource for future studies on endometriosis screening and treatment, especially for developing multi-organ segmentation methods for endometriosis.

## Methods

**Fig. 1:**
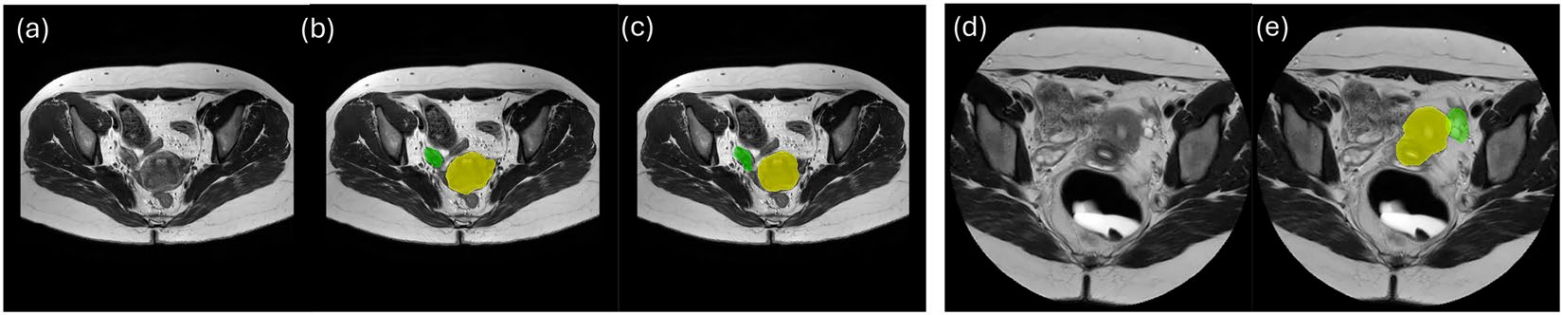
Examples of MRI scans for two Datasets. (**a**) T2-weighted MRI and (**b,c**) the corresponding uterus (yellow) and ovaries (green) labels from different raters for the first dataset. (**d**) T2-weighted MRI and (**e**) the corresponding uterus and ovaries labels for the second dataset.

This is a retrospective study. Our dataset comprises multi-sequence MRI scans collected from two clinical institutions in Texas: The Memorial Hermann Hospital System and Texas Children’s Hospital Pavilion for Women. Although both datasets were from patients suspected of having endometriosis, they include different MRI sequences obtained using varying protocols and MRI scanners. The study and data sharing policy allowing sharing for research purposes was approved by the Committee for the Protection of Human Subjects at UTHealth (protocol no HSC-SBMI-22-0184), which includes requirements for patient informed consent. Figure 1 shows examples of MRI scans and the corresponding labels for two Datasets.

### Image acquisition

Table 1 presents a summary of the two datasets collected in this study. The first dataset consists of MRI scans and labels for 51 patients before 2022. MR scans in this dataset were collected from 15 different sites using nine scanner models from three vendors (GE, Philips, and Siemens) with two magnetic field strengths (1.5 T and 3 T). Each site imaged a median of 3 subjects, ranging from 2 to 8. Each scanner model imaged a median of 4 subjects, ranging from 2 to 18. The MRI sequences include T2-weighted and T1-weighted fat suppression MRI. It is important to note that patients in this dataset were suspected of having endometriosis before undergoing MRI scans, resulting in eight patients who were not diagnosed with endometriosis. The second dataset comprises MRI scans and labels for 81 endometriosis patients from 2022. All the MRIs were taken in a single site with a Philips Ingenia 1.5 T MRI scanner. The MRI sequences in this dataset include T2-weighted, T2-weighted fat suppression, T1-weighted, and T1-weighted fat suppression MRI.

**Table 1:** Summary of Datasets in this Study.

Dataset	Clinical Institution (Sites, MR Scanner Models)	Application	Subjects (n)	Segmentation Target (n)	MRI sequences (Scans, Slices)	No. of Raters
1	Memorial Hermann Hospital System (15, 9)	Investigate Interrater Agreement	51	Uterus (49), Ovary (43), Endometrioma (40)	T1w (42, 3846) T1w FS (42, 3846) T2w (45, 1943)	3
2	Texas Children’s Hospital Pavilion for Women (1, 1)	Automatic Ovary Segmentation Pipeline	81	Uterus (62), Ovary (58), Endometrioma (11), Cyst (17)	T1w (76, 6675) T1w FS (68, 6006) T2w (50, 2439) T2w FS (77, 2785)	1

*T1w: T1-weighted, T2w: T2-weighted, FS: Fat Suppression.

### Data processing

Both datasets are de-identified by converting DICOM files to NIfTI format. The labels were manually contoured based on different MRI sequences by different raters in 3DSlicer. For the first dataset, three raters manually contoured structure segmentations for the uterus, ovaries, and endometriomas. The uterus and ovaries were contoured prioritizing based on T2-weighted sequences, while endometriomas were contoured prioritizing T1-weighted fat suppressed sequences. Note that in all cases, both T1 and T2 sequences were used. An experienced abdominal radiologist proposed a segmentation contouring guideline and reviewed the final labels from different raters to make necessary corrections. Manual segmentations from different raters are used to analyze interrater agreement. Each subject had one to two MRI sequences with two to four labels when the structures were present. Among the 51 subjects in Dataset 1, 11 (22%) were annotated by three raters, 22 (43%) by two raters, and 18 (35%) by one rater. For the second dataset, the uterus, ovaries, cysts, and endometriomas were manually contoured by an obstetrician-gynecologist assistant supervised by an expert Gynecologist, with all structures contoured based on T2-weighted fat suppression MRI with the same protocol. Although all patients in this dataset were diagnosed with endometriosis, only 12 had endometriomas.

Following patient enrollment and data collection, two specific analyses were conducted using the two datasets, respectively. Given that Dataset 1 was annotated by multiple raters, we used it to evaluate inter-rater agreement and to investigate how endometriosis may affect the segmentation accuracy of surrounding organs. For this analysis, seven subjects were selected from Dataset 1 based on the following inclusion and exclusion criteria. The inclusion criteria were as follows: (1) availability of a T2-weighted MRI; (2) suspected endometriosis; (3) manual segmentation of the ovaries and uterus by three raters. The exclusion criteria were as follows: (1) the segmentation was contoured by fewer than three raters; (2) ovarian segmentation covered obvious endometriomas or cysts.

Another key analysis involved developing an automatic ovary segmentation pipeline and evaluating its performance. Since all subjects in Dataset 2 were collected and annotated using a standardized protocol, this dataset was used to ensure consistency and reliability. A total of 38 subjects from Dataset 2 were included for pipeline development, with 30 cases used for training and validation, and 8 cases reserved for testing, based on the inclusion and exclusion criteria. The inclusion criteria were: (1) patients diagnosed with endometriosis and (2) availability of a T2-weighted fat suppression MRI with a corresponding manual ovary segmentation. The exclusion criteria were (1) patients with obvious endometriomas and (2) patients with cysts.

## Data Records

The raw data for each subject is available on Zenodo in NIFTI format^23^. The data from the first institution can be found in the /D1_MHS directory, where each subject’s subfolder contains registered MRI scans from different sequences and the corresponding labels contoured by multiple raters, identified by their rater IDs. The MR Scanner information is available in the /D1_MHS directory. The data from the second institution is in the /D2_TCPW directory, where each subject’s subfolder contains registered MRI scans and corresponding labels. Since these labels were contoured by a single rater, no rater ID is included in the second dataset.

## Technical Validation

### Interrater agreement analysis

**Fig. 2:**
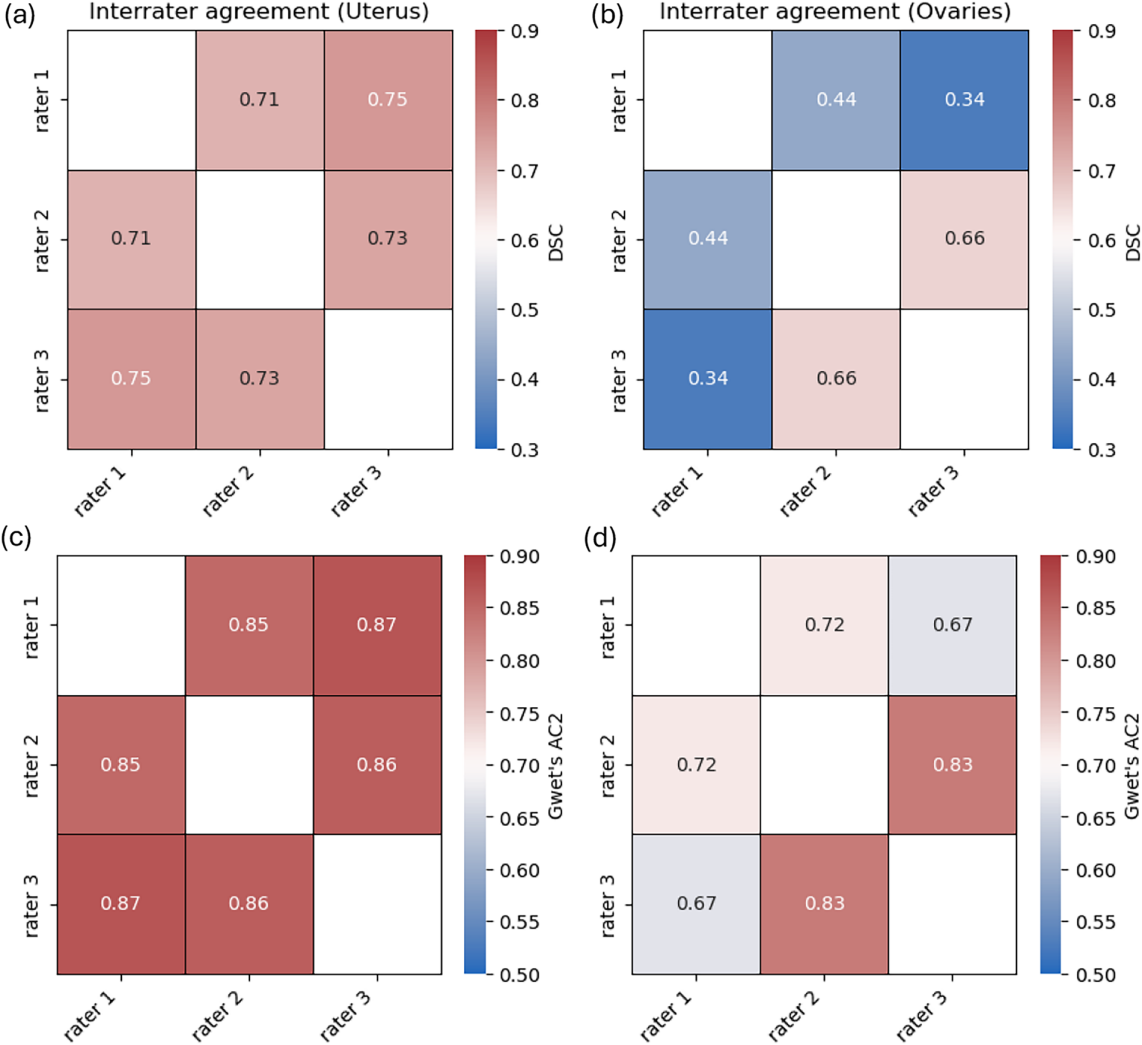
The Pairwise Interrater Agreement. Comparison of the pairwise DSC in (**a,b**) and pairwise Gwet’s AC2 (**c,d**) for Uterus (left) and Ovaries (right).

To assess interrater agreement for the uterus and ovaries as the two most critical surrounding anatomical structures affected by endometriosis using the first dataset, we use Krippendorff’s alpha at a nominal level based on the binary segmentation maps for each voxel to evaluate the reliability of segmentations provided by three raters^24^. Additionally, we assess pairwise interrater reliability using Gwet’s AC2^25^. Evaluation metrics are calculated for the uterus and ovaries to assess their segmentation quality. The interrater agreement was generally acceptable among all raters, with a Krippendorff’s α value of 0.73 for the uterus, while it was only 0.46 for the ovaries. The manual segmentations from three raters in the first dataset were evaluated by calculating the DSC. The average DSC value is 0.73 ± 0.18 for the uterus and 0.48 ± 0.24 for the ovaries. The average volume is 220.3 ± 120.2 cc for uterus segmentation and 12.2 ± 6.5 cc for ovaries segmentation. Table 2 shows the summary of the average performance for all raters. Compared with the uterus, the ovary in smaller volume shows lower DSC and lower interrater agreement for manual segmentation. Another measurement of interrater reliability also shows the same trend. The Gwet’s AC2 ranged from 0.85 to 0.87 with a median of 0.86 for the uterus and ranged from 0.67 to 0.83 with a median of 0.72 for the ovaries. Figure 2 shows pairwise agreement between all pairs of raters for two structures in the first dataset. Figure 2(a,b) show pairwise segmentation quality and similarity using DSC, and Fig. 2(c,d) show pairwise interrater agreement using Gwet’s AC2.

**Table 2:** Comparison of the Average Performance for Two Structures.

Structure	DSC	Krippendorff’s α	Volume (cc)
Uterus	0.73 ± 0.18	0.73	220.3 ± 120.2
Ovary	0.48 ± 0.24	0.46	12.2 ± 6.5

According to our interrater agreement analysis using Krippendorff’s α and Gwet’s AC2, we found that ovary segmentation had significantly lower interrater agreement compared to uterus segmentation, which has a larger volume and a more consistent location and shape. Additionally, Krippendorff’s α for ovary segmentation was less than 0.67, indicating moderate levels of agreement. Even under the guidance of an expert, different raters did not achieve a high level of agreement when contouring the ovary based on MRIs. This highlights the challenges of manual ovary segmentation and puts the segmentation performance of an automated ovary segmentation tool in perspective. Furthermore, evaluating inter-rater agreement for endometrioma segmentation will be considered as future work once additional subjects are enrolled.

### Auto-segmentation method

#### Data preprocessing

The selected subjects from the second dataset are used to develop the auto-segmentation method. The data were partitioned at the patient level into separate training, validation, and test sets to ensure subject-level independence across subsets. By developing our proposed auto-segmentation pipeline, we can demonstrate that our dataset is suitable for developing deep learning methods for medical imaging segmentation that could outperform the state-of-the-art methods. In the first step, we clipped the MRIs from the 1^st^ to the 99^th^ percentile and normalized them to a range of 0 to 1 for both datasets. For the auto-segmentation pipeline, the MRIs in dataset 2 are further preprocessed to enhance the representation of the ovaries, as described in the following equation.

$$I{\prime} (x)=\{\begin{array}{ll}{I}_{0}(x), & if\,{I}_{0}(x) < {o}_{1}\\ 1, & if\,{o}_{1}\le {I}_{0}(x) < {o}_{2}\\ {I}_{0}(x), & if\,{o}_{2}\le {I}_{0}(x) < 0.5\\ 1-{I}_{0}(x), & if\,{I}_{0}(x)\ge 0.5\end{array}$$

By analyzing the normalized dataset, we identified a range of intensity values, from a minimum intensity (*o*_1_) to a maximum intensity (*o*_2_), corresponding to the ovaries and related structures. For each voxel x, if its original intensity *I*_0_ falls within the range ${o}_{1}\le {I}_{0}(x) < {o}_{2}$, its intensity is set to 1 to highlight regions with features similar to those of the ovaries. The intensity is maintained if the original intensity of voxel x is less than 0.5 and outside the range (${o}_{1}$, ${o}_{2}$). For voxels with an original intensity greater than 0.5, the intensity is inverted to 1−*I*_0_ to reduce the impact of high-intensity values while preserving structural information. Analysis of the MRI scans in 3DSlicer revealed that the intensity of the ovaries in our dataset ranges from 0.22 to 0.3. In this study, we set ${o}_{1}=0.22$ and ${o}_{2}=0.3$ for all MRIs in the auto-segmentation pipeline to achieve optimal performance. This preprocessing method replicates the adjustments that radiologists typically make when reviewing MRI scans.

Data resampling and augmentation methods were applied at the slice level. Each slice was resampled to a height and width of 512 pixels, with a voxel size of 5 mm by 5 mm. Random translations within 25 pixels and rotations of up to 25 degrees were applied to increase the training dataset size by a factor of five.

#### Auto-segmentation pipeline

**Fig. 3:**
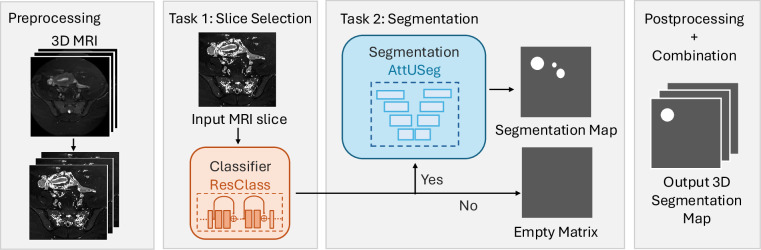
Overview of the RAovSeg for ovary auto-segmentation. The two core components are ResClass, a ResNet18-based classifier for selecting MR slices containing the ovary, and AttUSeg, an Attention U-Net-based segmentation model for creating 2D segmentation maps.

The overview of *RAovSeg* is illustrated in Fig. 3. Our auto-segmentation pipeline was trained on a single NVIDIA A100 GPU with 40 GB of memory using PyTorch 3.9. The first part of our method is the classifier, which we refer to as *ResClass*. It was trained on 2D MRI slices from all training subjects, utilizing 3,252 slices for training and 2,168 slices for validation. The model architecture is a two-layer 2D ResNet18 with 8 and 16 features in the respective layers. Binary Cross Entropy with Logits Loss (BCEWithLogitsLoss) was used to train the classifier.

To mitigate overfitting, we increased the size of the validation set, incorporated a dropout layer with a probability of 0.2, and applied L2 regularization. In the second step, the segmentation model, which we refer to as *AttUSeg*, was trained exclusively on MRI slices containing ovaries and their corresponding labels, comprising 594 MRI slices for training and 136 MRI slices for validation. This model was developed using a four-layer Attention U-Net architecture, with 16, 32, 64, and 128 features in each layer. The Focal Tversky Loss function, with parameters α = 0.8, β = 0.2, and γ = 1.33, was employed for training^26^. This loss function is particularly advantageous for segmenting small structures, such as the ovaries in our dataset, due to its ability to balance false positives and false negatives. After generating outputs from the segmentation model, two postprocessing methods—closing operation and connected component analysis—were applied to reduce false positive predictions.

#### Evaluation metrics

We use the Dice Similarity Coefficient (DSC) to evaluate segmentation quality. The interrater agreement is assessed using pairwise DSC calculations and the average DSC.

$${{DSC}}_{{avg}}=\frac{{{DSC}}_{12}+{{DSC}}_{13}+{{DSC}}_{23}}{3}$$

The average DSC is calculated using Equation 2, where DSC_ij_ represents the pairwise DSC between the segmentations contoured by rater i and rater j.

#### Quantitative analysis

Our quantitative analysis calculates the average DSC between the 3D segmentation outputs of all test subjects and their corresponding manual segmentations. The ablation study for *RAovSeg* is presented in Table 3. *RAovSeg* consists of the preprocessing, *ResClass*, *AttUSeg*, and postprocessing methods, achieving the highest DSC at 0.290. When the postprocessing method is removed, the remaining components, including our preprocessing method, *ResClass*, and *AttUSeg*, achieve a DSC of only 0.235. The DSC decreases further to 0.013 when *ResClass* is also removed, leaving only the preprocessing methods and *AttUSeg*. This result highlights the critical importance of *ResClass* in our pipeline. We also evaluated the impact of our proposed preprocessing and postprocessing methods on the performance of nnU-Net. The results of this ablation study are presented in Table 4. We used the 3D full-resolution nnU-Net to generate the ovary segmentation map, resulting in a DSC of 0.272. For comparison, we also applied our proposed preprocessing and postprocessing methods to the uuU-Net framework. However, these methods did not lead to noticeable improvement in U-Net’s performance, yielding DSC values of 0.267 and 0.200, respectively. The *RAovSeg* achieved a DSC of 0.290 for ovary segmentation, which outperforms nnU-Net.

**Table 3:** Ablation study for RAovSeg.

Ablation Study for Our Proposed Pipeline	
Component(s)	DSC
RAovSeg*	**0.290**
Preprocessing + ResClass + AttUSeg	0.235
Preprocessing + AttUSeg	0.013

*RAovSeg consists of Preprocessing, ResClass, AttUSeg, and Postprocessing.

**Table 4:** Ablation study for nnU-Net.

Component(s)	DSC
Preprocessing + nnU-Net + Postprocessing	0.200
Preprocessing + nnU-Net	0.267
nnU-Net	0.272

#### Qualitative analysis

**Fig. 4:**
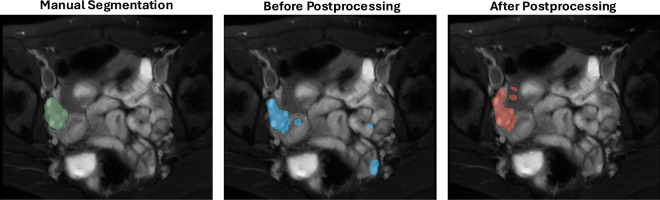
Example of the postprocessing method to reduce the false positives. This is the comparison among the manual segmentation (in green), the segmentation results before (in blue), and after postprocessing (in red). The segmentation results after postprocessing show fewer false positives.

**Fig. 5:**
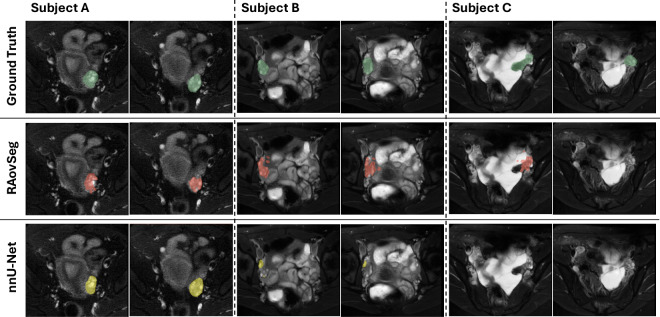
The comparison of the segmentation results for our method and nnU-Net for Subjects A, B, and C. Each subplot shows the manual segmentation (first row in green), the segmentation results from RAovSeg (second row in red), and the segmentation results from nnU-Net (third row in yellow) for different subjects. Each column is for the same slices.

Figure 4 shows an example of the segmentation results before and after applying the postprocessing method. Compared to the segmentation results before postprocessing, the results after postprocessing have fewer false positives in the bottom right region, which corresponds to the intestinal tract. However, postprocessing increases the number of false positives near our segmentation target, the ovary, due to the influence of adjacent slices. Although the postprocessing method significantly enhances segmentation performance by reducing false positives, some false positives remain in some cases. Figure 5 shows the segmentation results for three subjects. For Subject A, both our proposed method and nnU-Net successfully detect the ovary’s position and shape. They achieve comparable performance. For Subject B, our proposed method precisely predicts the ovary’s position, size, and shape, though some false positives are generated. In contrast, nnU-Net predicts only parts of the ovary with a much smaller volume than manual segmentation. In Subject C, the ovary has an irregular shape. Our method detects the ovary with an inaccurate shape in one of the two slices shown in this figure, while nnU-Net fails to predict the ovary in this subject.

In this study, our experiments demonstrate that a customized deep learning method can generate promising results that surpass the performance of the baseline model, nnU-Net. By utilizing this dataset, further work can contribute to the improvement of absolute segmentation performance and its integration into a complete imaging pipeline for endometriosis screening and tracking.

## Data Availability

The software employed in this study for data generation or processing is described in the Methods section. No custom code was used to curate or validate the dataset itself. The image analysis tools developed for RAovSeg are now publicly accessible on GitHub at: https://github.com/xlianguth/RAovSeg.
